# Postoperative pain and pain-related health-care contacts after open inguinal hernia repair with Adhesix™ and Progrip™: a randomized controlled trial

**DOI:** 10.1007/s10029-021-02549-8

**Published:** 2022-01-22

**Authors:** A.-M. Thölix, J. Kössi, J. Harju

**Affiliations:** 1 Department of Abdominal Surgery, University of Helsinki and Helsinki University Hospital, Helsinki, Finland; 2 Department of Surgery, Päijät-Hämeen keskussairaala, Lahti, Finland

**Keywords:** Inguinal hernia, Mesh fixation, Open repair

## Abstract

**Purpose:**

Self-fixed mesh is an alternative to suture mesh fixation in inguinal hernia repair. The aim of this study was to evaluate postoperative pain after open inguinal hernia surgery using self-fixed meshes.

**Methods:**

A randomized clinical trial comparing self-adhesive mesh (Adhesix™) and self-gripping mesh (Progrip™) was conducted from November 2018 through March 2021. Patients included were male, 18–85 years old, and suitable for day case surgery. The primary endpoint was the number of patients needing follow-up visits due to postoperative pain during the first 3 postoperative months. Secondary endpoints included the intensity of pain, the time of return to work and normal daily activities, quality of life measures and postoperative complications.

**Results:**

270 patients were enrolled, 132 received Adhesix™ mesh (A group) and 138 Progrip™ mesh (P group), 231 (85.6%) completed 1- or 3-month follow-up. The number of patients needing follow-up for postoperative pain was significantly higher in the P group (19 vs. 4, *p* = 0.001). The P group had higher numeric rating scale of pain while coughing (P 0.50 vs. A 0.20, *p* = 0.024) and during exercise (P 1.02 vs. A 0.60, *p* = 0.057) at 3 months postoperatively. The time of return to normal activity was 16.6 days in the A group and 22.9 days in the P group, (*p* = 0.004). The postoperative day being fit for work was sooner for the A group (14.3 days vs 17.8 days, *p* = 0.009).

**Conclusion:**

This study demonstrated an advantage of self-adhesive mesh over self-gripping mesh with respect to acute postoperative pain and thus faster recovery after surgery.

## Introduction

Inguinal hernia repair is one of the most performed surgical operations [1]. For decades, the gold standard for inguinal hernia repair has been the tension-free Lichtenstein technique, with the use of a sutured non-absorbable mesh [2]. This technique is safe, with a low complication and recurrence rate [1]. An alternative to suturing the mesh is using glue fixation, a self-gripping mesh, or a self-adhesive mesh. Several previous analyses [3–7] have shown that sutureless fixation has the same outcome in postoperative complications and recurrence rate.

The self-gripping mesh (Progrip™) consists of resorbable microgrips fixing the mesh [7], whereas the self-adhesive mesh (Adhesix™) is impregnated with a self-adhering gel of polyvinylpyrrolidone (PVP) and polyethylene glycol (PEG) [3].

Today, the most frequent problem after hernia repair is chronic inguinal pain [1, 8, 9]. The pain incidence varies in the literature and approximately 10–21% of operated patients suffer from chronic pain [8–10], usually decreasing with time after implantation. The cause of chronic pain is uncertain, but various risk factors have been noted in earlier studies, such as open procedure compared to laparoscopic surgery, female patients, patients of younger age, preoperative pain, and severe early postoperative pain [7–11]. Light-weighted mesh is associated with less pain [10] and surgery for recurrent hernia increases the risk of chronic pain [8, 11]. There seems to be no difference between mesh fixation technique and chronic pain, according to two quite recent meta-analyses [6, 12], where self-gripping mesh, adhesional fixation and sutured mesh had equal rates of chronic pain. The present study was designed to compare self-adhesive mesh with Progrip™ self-gripping mesh in preventing early postoperative pain.

Another important aspect is acute pain affecting the return to work and daily activities as well as affecting the requirement for the use of pain medication after the operation. Previous studies indicate less early postoperative pain when using glue fixation for the mesh [4, 5, 12, 13]. Two randomized clinical trials [4, 13] described diminished postoperative pain up to one month after the operation with glue fixation compared to sutured mesh. The self-gripping mesh also reduced early postoperative pain at 1 week compared to suture fixation according to another RCT [14], but at 1 month or later there was no difference in pain. A quite recent meta-analysis [12] reviewed significantly lower mean VAS scores (visual analog scale for pain) at 1 week and 1 month after surgery with adhering or self‐gripping fixation methods compared to suture fixation. Although the self-adhesive mesh is used widely, there are only few small trials examining self-adhesive meshes. According to a retrospective study [15], the number of patient contacts due to postoperative pain was less common when using a self-adhesive mesh than with the self-gripping mesh, which may indicate less postoperative pain.

Several published trials and meta-analyses comparing self-gripping mesh, glue fixation and sutured mesh, have indicated that glue fixation seems to cause less pain in the early phase after the operation [4, 5, 12–14]. However, to our best knowledge, there are no randomized controlled trials evaluating the differences between self-adhesive and self-gripping mesh.

The aim of this trial was to evaluate postoperative pain after open inguinal hernia surgery using two different self-fixed meshes. The hypothesis for the study was that the use of a non-traumatic sutureless self-adhesive mesh might reduce early postoperative pain compared to a self-gripping mesh.

## Methods

### Study design

A randomized clinical trial comparing two different self-adhering meshes in open hernia repair was conducted from November 2018 through March 2021. The study was approved by the national ethics committee of Helsinki University hospital (HUS/459/2018) and registered in ClinicalTrails.com (NCT03734224). Participants were recruited from two surgical units in Finland, Helsinki University Hospital and Päijät-Häme Central Hospital.

### Inclusion criteria

The enrolled subjects were male, aged between 18 and 85 years and suitable for day case surgery. All subjects had a symptomatic primary unilateral inguinal hernia confirmed by clinical examination.

**Fig. 1:**
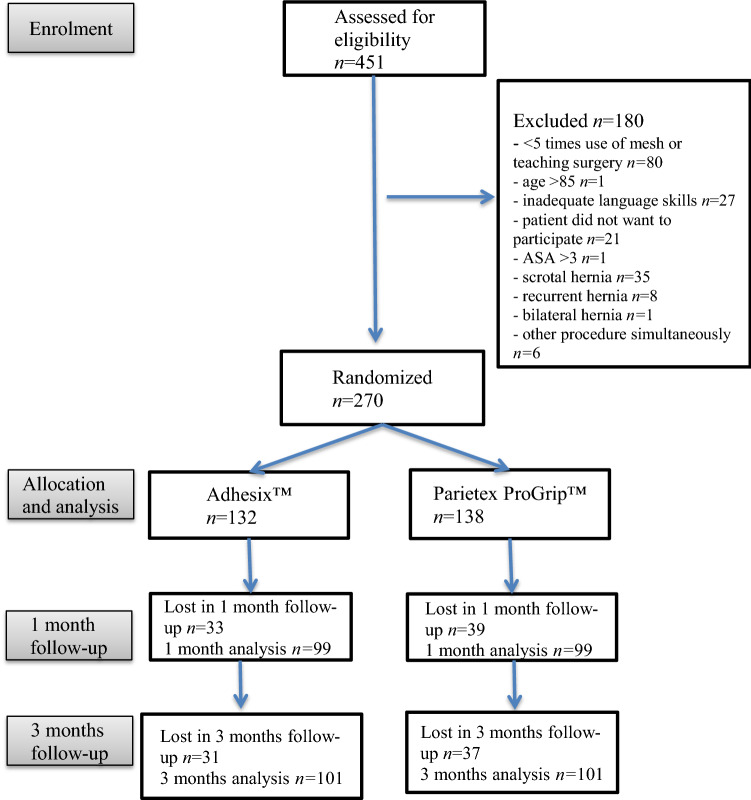
Flowchart of the study design

The study excluded subjects with a bilateral, recurrent, or incarcerated hernia, an American Society of Anaesthesiologists physical status (ASA) of IV or more and a body mass index higher than 35 or lower than 18. The patients were also excluded if the surgeon was not experienced with both meshes or due to teaching surgery (surgical trainee performing part of whole operation under supervision of a consultant). An experience of both meshes at least five times was required. The patients were also excluded if they did not agree to participate or had inadequate language skills. The excluded patients were recorded, Fig. 1*.*

The eligible subjects received oral and written information about the study. Each participant signed an informed consent.

The operating surgeons (*n* = 8) were experienced in open hernia surgery.

### Randomization

Patients were enrolled consecutively and randomly allocated to receive one of the treating options: the self-adhesive mesh (Mesh Adhesix™, 7.5 cm × 15.5 cm, Cousin Biotech, France) or self-gripping mesh (Progrip™ Self-gripping polypropylene mesh, 12 cm × 8 cm, Covidien) in an otherwise similar open surgical procedure. Randomization was stratified in groups for each center, with block randomization of 10. Numbered and sealed opaque envelopes were opened by the operating surgeon just before the procedure.

### Operative technique

The operation was performed using local or general anaesthesia, with preference of local anaesthesia. The local anaesthetics (Ropivacaine) was administered by the operating surgeon. General anaesthesia was used only if the local anaesthesia was insufficient or at patient request. An inguinal skin incision was made, the external oblique aponeurosis was divided, the inguinal canal was exposed through an open anterior approach and the spermatic cord was dissected free. Direct hernia was inverted, and indirect hernia sacs were dissected and resected. The regional nerves were preserved when possible. The mesh was placed on the inguinal ligament, the pubic bone and the internal oblique aponeurosis. No additional fixation was used. The external oblique aponeurosis, Scarpa fascia and skin were closed.

### Primary and secondary outcomes

The primary endpoint was the number of patients needing unplanned visits to a physician in the health-care center (operating hospital or public and private health-care centers) due to postoperative pain during the first 3 months after the operation. Secondary endpoints included use of pain medication, the intensity of pain (measured by the Numeric rating scale, NRS, ranging from 0 to 10), the time of return to work and normal daily activities, quality of life measures (measured by RAND-36 scale [16]) and postoperative complications.

### Patient data

Age, weight, height, body mass index, ASA classification and use of anticoagulants were recorded. The patients reported intensity of pain in various situations and the use of pain medication before the operation. The physical requirements of work were inquired of the patient. Operation time, the type of mesh, the type of anaesthesia, hernia type and possible nerve resection were recorded.

Clinical examination was conducted before the operation. Inquiries were collected by the surgeons at 1 and 3 months after the operation and the patient´s medical records were reviewed postoperatively. The NRS pain scale from 0 to 10 were assessed at rest, when coughing and during physical activity before surgery and at one and 3 months after the operation. The NRS scale is a simple and commonly used pain measurement tool, from 0 to 10, where 0 being “no pain” and 10 “the worst pain imaginable”. To assess quality of life outcomes the RAND-36 item health survey was used preoperatively and at follow-up 1 and 3 months after surgery. The patients continuously followed-up and reported use of regular and intermittent pain medication, the postoperative day of return to normal physical activity and the day of being fit for work during the postoperative inquiries. Complications and number of follow-up visits due to pain or other problems were recorded. Additional evaluations at the outpatient clinic were performed only in patients with pain or complications needing treatment.

### Statistical analysis

Prior to this study, sample size estimation based on our earlier published retrospective study (14), indicated that approximately 216 patients should be included in each group to detect clinically relevant difference between the outcomes (*α* 0,05, *β* 0,80). We aimed for 480 patients in total, to allow a 10% dropout rate. An interim analysis was conducted halfway as planned, and the recruitment of the patients was terminated at this point due to significant difference between the groups in primary outcome.

Variables were tested for normality of distributions with the Kolmogorov–Smirnov test. Comparison of numeric data between the two groups were performed with independent samples *t* test or Mann–Whitney *U* test, depending on whether the variables followed a normal or non-normal distribution. The Chi-square test or Fisher´s exact test was used for comparisons of categorical data.

Data are presented as mean (standard deviation) for numeric data and as frequency for categorical variables. A value of *p* < 0.05 was considered as statistically significant.

All statistical analyses were performed using IBM SPSS statistics version 27.

## Results

In June 2021, a formal interim analysis for the primary endpoints was performed. Data of 270 patients were reviewed, 231 (85.6%) of whom had completed a 1- or 3-month follow-up. The response rate for the 1-month follow-up was 73.3% and 74.5% for the 3-month follow-up. The recruitment of patients was discontinued at this stage due to the findings and the results obtained were analyzed. Here, we publish the data of the 270 patients who were included in the interim analysis.

### Patient characteristics

A total of 270 patients were randomly assigned into two groups between November 2018 and March 2021, 132 of whom received Adhesix™ mesh (A group) and 138 Progrip™ mesh (P group). Table 1 shows baseline characteristics, which were similar between the two groups.

**Table 1:** Baseline characteristics

Characteristics	Adhesix™ (*n* = 132)	Progrip™ (*n* = 138)	Total (*n* = 270)	*p* value
Age (years), mean (SD)	54.5 (15.2)	55.4 (14.7)	54.9 (14.9)	0.607 *
BMI (kg/m^2^), mean (SD)	24.5 (2.6)	25.2 (3.2)	24.9 (3.0)	0.052 *
ASA classification, *n* (%)				
I	79 (60.3%)	76 (55.5%)	155 (57.8%)	0.684 ^2^
II	44 (33.6%)	53 (38.7%)	97 (36.2%)	
III	8 (6.1%)	8 (5.8%)	16 (6.0%)	

*p*-value: ^2^Chi-square test, *Student´s *T* test

### Operating details

The two groups had similar operating details, as seen in Table 2, except for the type of anaesthesia. In the P group a significantly higher number of the operations were performed under general anesthesia (*p* = 0.007). No intra-operative complications were observed. The mean operation time was 42 min in both groups. Nerve resection was performed in 18 (6.7%) surgeries, with comparable results for both groups.

**Table 2:** Operative details

Operative details	Adhesix™ (*n* = 132)	Progrip™ (*n* = 138)	Total (*n* = 270)	*p* value
Anesthesia, *n* (%)				
Local	109 (83.2)	95 (68.8)	204 (75.8)	**0.007 **^**2**^
General	22 (16.8)	43 (31.2)	65 (24.2)	
Hernia type, *n*				
Direct (M1/M2/M3)	52 (5/41/6)	46 (6/37/3)	98	0.475 ^2^
Indirect (L1/L2/L3)	63 (33/29/1)	81 (42/39/0)	144	
Combined (Pantaloon hernia)	16	11	27	
Nerve resection, *n (%)*				
Iliohypogastric	11 (8.3)	7 (5.1)	18 (6.7)	0.276 ^2^
Ilioinguinal	2 (1.5)	1 (0.7)	3 (1.1)	
Genitofemoral	0 (0)	2 (1.4)	2 (0.7)	
None	119 (90.2)	128 (92.8)	247 (91.5)	
Operation time (min), mean (SD)	42.31 (11.710)	42.20 (12.290)		0.936*

The bold values are statistically significant p-values

*p*-value: ^2^Chi-square test, *Student´s *T* test

### Primary outcome

The primary outcome was the number of patients needing follow-up visits in the health-care center due to postoperative pain in the inguinal area during the first 3 months after the operation, which was significantly higher in the P group (*n* = 19 vs. *n* = 4, *p* = 0.001). During the first month of follow-up 14 (14.3%) patients in the P group had a follow-up visit at a doctor´s appointment due to pain, while during the following 2 months up to 3 months after surgery an additional of five patients in the P group needed an evaluation for their pain. The equivalent result for patients in the A group was 2 (2.1%) and 2 (2.0%), respectively. The difference between the groups was significant during the first month (*p* = 0.002), but not after that (*p* = 0.149) Table 3*.*

**Table 3:** Early pain measures

Pain measures			*p* value
Preoperative	Adhesix™ (*n* = 132)	Progrip™ (*n* = 138)	
Number of patients using pain medication preoperatively, *n* (%)	28 (21.5%)	28 (21.1%)	1.00º
Daily, *n* (%)	3 (2.3%)	2 (1.5%)	
Weekly, *n* (%)	6 (4.6%)	7 (5.3%)	
Sometimes, *n* (%)	19 (14.6%)	21 (15.8%)	
Never, *n* (%)	102 (78.5%)	103 (77.4%)	

1 month postoperative	Adhesix™ (*n* = 99)	Progrip™ (*n* = 99)	
Return to normal activity (postoperative days), mean (SD)	16.6 (14.0)	22.9 (16.0)	0.004 *
Fit for work (postoperative days), mean (SD)	14.3 (7.7)	17.8 (9.4)	0.009 *
Use of regular pain medication (postoperative days), mean (SD)	7.4 (7.0)	9.3 (7.9)	0.081 *
Use of intermittent pain medication (postoperative days), mean (SD)	11.1 (12.1)	13.5 (13.9)	0.215 *
Follow-up visits due to pain 0–1 month after surgery (*n*/%)	2/2.1%	14/14.3%	**0.002 **^**2**^

3 months postoperative	Adhesix™ (*n* = 101)	Progrip™ (*n* = 101)	
Follow-up visits due to pain > 1–3 month after surgery (*n*/%)	2/2.0%	6/6.0%	0.149 ^2^
All follow-up vistits due to pain 0–3 months after surgery (*n*/%)	4/3.2%	19/14.7%	**0.001 **^**2**^

The bold values are statistically significant p-values

*p*-Value: ^2^Chi square, ºFischer, *Student´s *t*-test

When comparing patients using pain medication preoperatively for inguinal pain with those who did not need pain medication, the number of pain-related follow-up visits were equal (*p* = 0.803) and there was no difference in the number of patients with more severe pain in both groups.

### Secondary outcomes

#### Measures of pain

The degree of pain was estimated preoperatively and at 1- and 3-month surveys with numeric pain scores (NRS) assessed at rest, when coughing and during physical activity. The preoperative NRS score was higher (4.81) in the P group during exercise, compared to the A group (4.09), *p* = 0.041. At the 3-month follow-up, the patients in the P group had more pain while coughing (*P* 0.50 vs. A 0.20, *p* = 0.024) and during exercise (*P* 1.02 vs. A 0.60, *p* = 0.057). Within the groups, NRS assessed at rest was not yet significantly improved at 1 month compared to the preoperative situation, however, at 3 months, most patients had significantly lower NRS. NRS when coughing or during physical activity showed distinct improvement at 1 month compared to the preoperative situation, continuing at 3 months with even lower numbers in both groups, as shown in Table 4.

**Table 4:** Numeric pain scores, NRS 0–10

NRS scale	Adhesix™	Progrip™	*p* value
Preoperative NRS at rest	0.79 (1.456)	0.97 (1.574)	0.229ʷ
Preoperative NRS when coughing	2.36 (2.455)	2.66 (2.519)	0.261ʷ
Preoperative NRS at exercise	4.09 (2.777)	4.81 (2.782)	**0.041ʷ**
1-month NRS at rest	0.48 (1.248)	0.65 (1.185)	0.217ʷ
1-month NRS when coughing	0.83 (1.506)	1.06 (1.682)	0.406ʷ
1-month NRS at exercise	1.42 (1.796)	1.67 (1.815)	0.205ʷ
3-month NRS at rest	0.19 (0.817)	0.23 (0.604)	0.196ʷ
3-month NRS when coughing	0.20 (0.903)	0.50 (1.286)	**0.024ʷ**
3-month NRS at exercise	0.60 (1.285)	1.02 (1.578)	0.057ʷ

NRS change within the groups	Preoperatively	1 month	Mean change (95% CI)	*p* value
A group; NRS at rest	0.77 (1.505)	0.48 (1.254)	0.284 (− 0.059 to 0.628)	0.104°
P group; NRS at rest	0.97 (1.612)	0.67 (1.194)	0.302 (− 0.052 to 0.656)	0.094°
A group; NRS when coughing	2.31 (2.368)	0.85(1.518)	1.462 (0.253–0.959)	** < 0.001°**
P group; NRS when coughing	2.55 (2.525)	1.07 (1.687)	1.479 (0.919–2.039)	** < 0.001°**
A group; NRS at exercise	3.99 (2.621)	1.44 (1.799)	2.553 (2.013–3.094)	** < 0.001°**
P group; NRS at exercise	4.55 (2.937)	1.69 (1.816)	2.856 (2.152–3.559)	** < 0.001°**

NRS change within the group	Preoperatively	3 months	Mean change (95% CI)	*p* value
A group; NRS at rest	0.77 (1.505)	0.19 (0.813)	0.460 (0.209–0.711)	** < 0.001°**
P group; NRS at rest	0.97 (1.612)	0.23 (0.609)	0.653 (0.325–0.981)	** < 0.001°**
A group; NRS when coughing	2.31 (2.368)	0.20 (0.903)	1.949 (1.509–2.390)	** < 0.001°**
P group; NRS when coughing	2.55 (2.525)	0.51 (1.292)	1.866 (1.363–2.369)	** < 0.001°**
A group; NRS at exercise	3.99 (2.621)	0.60 (1.279)	3.280 (2.758–3.802)	** < 0.001°**
P group; NRS at exercise	4.55 (2.937)	1.03 (1.582)	3.194 (2.607–3.779)	** < 0.001°**

The bold values are statistically significant p-values

Mean (SD). *p*-Value: °Paired-samples *T* test, ʷMann–Whitney-*U*

The use of regular and intermittent pain medication postoperatively was comparable between the groups. The patients were routinely prescribed paracetamol and ibuprofen. Only a minority of the patients used pain medications more than 1 month postoperatively (A *n* = 6/6.1%, *P* *n* = 12/11.9%).

20.2% of the patients in the P group versus 9.0% in the A group had not returned to normal activity at 1 month postoperatively, the number was significantly higher in the P group (*p* = 0.025). According to patients´ own estimate, the mean time of return to normal activity was 16.6 days in the A group and 22.9 days in the P group, (*p* = 0.004). Additionally, the postoperative day being fit for work was sooner for the A group (14.3 days vs 17.8 days, *p* = 0.009), Table 3.

### Complications

27 (13.7%) patients had complications that needed a doctor’s evaluation or treatment at the outpatient clinic. 3.6% had a postoperative infection treated with antibiotics, one patient suffered from a large postoperative haematoma, which was evacuated on the first postoperative day. Other reasons were superficial wound problems, i.e. haematomas and swelling in the operated area. In the inquiries, patients were asked to report clinical findings after the operation. Beyond complications mentioned above, superficial skin bruising was common among the patients, 36.4% reported bruising in the inguinal region and 49.5% in the scrotum. None of the patients had a recurrence during the first 3 months. Table 5 shows the number of complications in each group.

**Table 5:** Postoperative complications, *n* (%)

Complications	Adhesix™ (*n* = 99)	Progrip™ (*n* = 99)	Total (*n* = 198)	*p* Value
Follow-up visits due to complications	14 (14.1)	13 (13.3)	27 (13.7)	0.858
Reoperation due to haematoma	1 (1.0)	0 (0)	1 (0.5)	0.316
Inguinal skin bruising	39 (39.4)	33 (33.3)	72 (36.4)	0.375
Scrotal skin bruising	54 (54.5)	44 (44.4)	98 (49.5)	0.155
Surgical site infection	4 (4.1)	3 (3.0)	7 (3.6)	0.690
Superficial	3	3	6	
Deep	1	0	1	
Reoperation for mesh removal	0	0	0	
Seroma/wound swelling	18 (18.6)	12 (12.6)	30 (15.6)	0.258
Recurrent hernia	0 (0)	0 (0)	0 (0)	

*p*-Value: Chi-square test

#### RAND-36

**Fig. 2:**
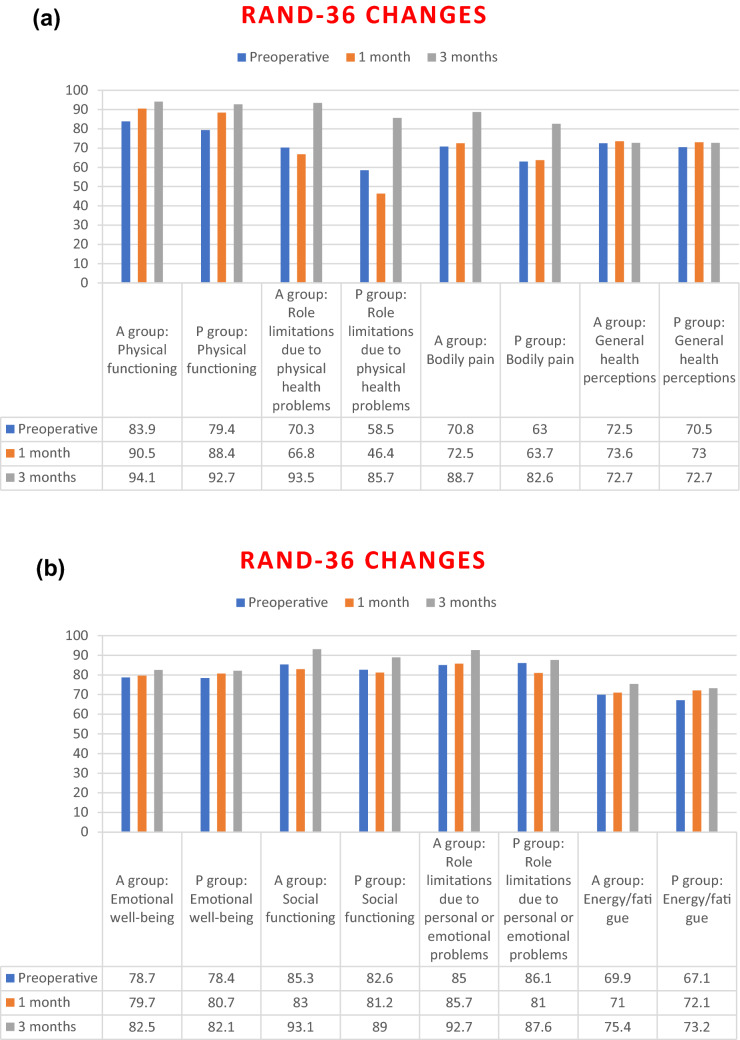
RAND-36 change preoperatively and at 1 and 3-month follow-up. **a** physical functioning and role limitations, pain and general health measurements. **b** emotional and social functioning and role limitations

The RAND 36-Item Health Survey [16] contains eight health concepts: physical functioning, role limitations due to physical health problems (during the last 4 weeks), role limitations due to personal or emotional problems (during the last 4 weeks), energy/fatigue, emotional well-being, social functioning, bodily pain, and general health perceptions. Each item is scored on a 0–100 range so that the lowest and highest possible scores are 0 and 100, respectively. Scores represent the percentage of total possible score achieved. The mean scores and changes are presented in Fig. 2*.*

Physical functioning was improved in both groups at 1 and 3 months compared to the preoperative situation. However, role limitations due to physical health problems in the P group was significantly inferior at one month compared to the preoperative state, 46.4 and 58.8, respectively (*p* = 0.026). The same decrease was not seen in the A group. Neither group had any change in pain at 1 month, yet at 3 months patients in both groups experienced less pain (*p* < 0.001).

## Discussion

To our knowledge, this is the first study comparing self-adhesive and self-gripping meshes in a randomized setting. This trial showed that in a short follow-up time of 3 months, a self-adhesive mesh caused less early postoperative pain compared to a self-gripping mesh, with otherwise comparable results. Here, we focused on a clinically relevant endpoint, that is the number of patients needing additional follow-up due to postoperative pain which was significantly higher in the P group (19 vs. 4, *p* = 0.001), particularly during the first postoperative month. This finding is in line with our retrospective study showing that the use of self-adhesive mesh was associated with less postoperative pain compared to self-gripping mesh [15].

As pain is difficult to estimate reliably, we used several different ways to measure it. Pain according to NRS-scores improved postoperatively in both groups, with low level of pain at 3 months. Moreover, the A group had significantly lower scores during movement at this endpoint. Likewise, quality of life assessments (RAND-36) indicated some better results for the self-adhesive mesh. These patients also recovered more rapidly when considering return to work and other daily activities. On the other hand, no difference in the amount of pain medication after surgery was observed between the groups.

In earlier analyses [5, 8, 12, 14, 17], the difference between glue fixation and self-gripping mesh seems to be indistinct. A meta-analysis [12] including 5190 patients comparing self-gripping mesh and mesh fixation using glue with sutured mesh demonstrated no difference in chronic pain, but short-term postoperative pain favoured a non-sutured technique. Their subgroup analysis established a significant reduction of the VAS score when using glue compared to sutures at 1 week and 1 month after surgery, but the same result failed between self-gripping mesh and sutured mesh. This result supports our finding in this clinical trial. Furthermore, another meta-analysis [5] showed a lower VAS score at day 1 for the glue fixation group compared to self-gripping mesh, but no difference at 1 week or later.

Additionally, we are aware of the fact that glue fixation and self-adhesive mesh consisting of a glue surface are not totally equal, even though glue is the fastener in both. But, as there are currently no clinical trials comparing self-adhesive mesh to self-gripping mesh or any other kind of fixation, we suggest that glue fixation is the closest comparable type. As to the evaluation of self-adhesive mesh (Adhesix™) applied in this study, earlier studies report low pain and complication rates [3, 15] for Adhesix™.

Severe early postoperative pain is a predictive factor for developing chronic pain, as shown in a randomized clinical trial [8] with 625 patients comparing sutured mesh, self-gripping mesh, and glue-fixated mesh. In this context, our result shows clinical relevance. Moreover, an earlier return to normal activities is not only important for the patient, but also for society, with lower costs. Naturally, many factors may influence this result; however, we evaluated these factors as minor in this study, as patient characteristics in the two groups were comparable for other risk factors such as preoperative pain rates or patient age.

One explanation for more severe pain in the early postoperative time in the self-gripping group might be the microgrips on the surface of the self-gripping mesh, which had not resorbed by the time of follow-up. The resorption time of microgrips are 12 months [7]. Furthermore, the self-adhesive mesh may cause less irritation in the surrounding tissue and less damage to inguinal nerves [17], and thereby induce less pain. A Cochrane database systematic review [18] also indicates that glue fixation may reduce postoperative pain. Another possible factor causing more postoperative pain in the P group might be the type of hernia. A randomized clinical trial demonstrated a significant benefit from the omission of high hernia sac ligation and excision on postoperative pain on patients who undergo tension-free indirect inguinal hernia repair [19]. In this trial, the operative technique included hernia sac dissection and ligation in indirect hernias. Although the distribution of hernia types in our study was not statistically significantly different (*p* = 0.475), the actual number of indirect hernias was higher in self-gripping mesh group (58.0%) compared to the self-adhesive mesh group (47.7%) which may have effect on our results.

Previous papers [4–8, 12] have shown no difference in chronic pain between the different mesh fixation methods and no conclusions regarding chronic pain can be made based on our current study due to the short follow-up time. However, our study is continuing and long-term results will be published later.

Apart from pain-related problems, this study demonstrated a low complication rate during or after surgery in the short follow-up time of 3 months. This confirms the findings of earlier studies [4, 5, 13, 18] showing that open hernia surgery is a safe procedure. However, according to our inquiries, many patients reported minor, not clinically relevant disadvantages, such as inguinal and scrotal bruising or fluid collection. However, the need for treatment due to complications was scarce. No hernia recurrence was diagnosed during this short follow-up.

This study has some limitations. First, it is well known that acute pain is subjective and difficult to measure reliably. Many factors influence the sensation of pain. Second, the patients were not routinely checked up after surgery, except for the symptomatic patients needing an evaluation. Third, response rate of the postoperative questionnaires could have been higher but, however, the response rate was similar for both groups.

The strength of this study is the randomized clinical design and the follow-up of the outcomes of patients suitable for day-case surgery during the first 3 postoperative months. This provides reliable results on pain and its impact on daily life during the early postoperative phase.

### Conclusion

In summary, the present study demonstrated an advantage of self-adhesive mesh over self-gripping mesh with respect to acute postoperative pain and thus faster recovery after surgery. Both meshes had low and comparable complication rates.
